# Preterm‐born individuals: a vulnerable population at risk of cardiovascular morbidity and mortality during thermal extremes?

**DOI:** 10.1113/EP091152

**Published:** 2023-04-21

**Authors:** Ryan Phillip Sixtus, Clint Gray, Mary Judith Berry, Rebecca Maree Dyson

**Affiliations:** ^1^ Department of Paediatrics and Child Health University of Otago Wellington New Zealand

**Keywords:** cardiovascular, cardiovascular dysfunction, preterm birth, thermal extremes, thermoregulation, vulnerable populations

## Abstract

Preterm‐born individuals are a uniquely vulnerable population. Preterm exposure to the extrauterine environment and the (mal)adaptations that occur during the transitional period can result in alterations to their macro‐ and micro‐physiological state. The physiological adaptations that increase survival in the short term may place those born preterm on a trajectory of lifelong dysfunction and later‐life decompensation. Cardiovascular compensation in children and adolescents, which masks this trajectory of dysfunction, is overcome under stress, such that the functional cardiovascular capacity is reduced and recovery impaired following physiological stress. This has implications for their response to thermal stress. As the Anthropocene introduces greater changes in our environment, thermal extremes will impact vulnerable populations as yet unidentified in the climate change context. Here, we present the hypothesis that individuals born preterm are a vulnerable population at an increased risk of cardiovascular morbidity and mortality during thermal extremes.

## Abstract (highlights)

- **What is the topic of this review?**
  Thermal extremes disproportionately affect populations with cardiovascular conditions. Preterm birth, across all gestational age ranges below 37 weeks, has been identified as a non‐modifiable risk factor for cardiovascular disease. The hypothesis is presented that individuals born preterm are at an increased risk of cardiovascular morbidity and mortality during thermal extremes.
- **What advances does it highlight?**
  Cardiovascular stress tests performed in preterm‐born populations, from infancy through adulthood, highlight a progression of cardiovascular dysfunction accelerating through adolescence and adulthood. This dysfunction has many similarities with populations known to be at risk in thermal extremes.

## 1 INTRODUCTION

Across the first decade of the 21st century, heat‐related morbidity and mortality rose 20% above the previous decade (K. R. Smith et al., 2014). This trend of extreme thermal events, and associated mortality, has increased in both frequency and severity across the second decade (Fischer & Knutti, 2015). In 2015, global temperatures rose 0.85°C above the 20th century average, contributing to a four‐ to five‐fold increase in heatwaves, with this increase predominantly affecting the most rare and extreme precipitation and heatwave events (Fischer & Knutti, 2015). As of 2020, global land and ocean temperatures were 0.98 (±0.15)°C above the 20th century average, with each successive 5‐year period exceeding the last (World Meteorological Organization, 2019).

**FIGURE 1:**
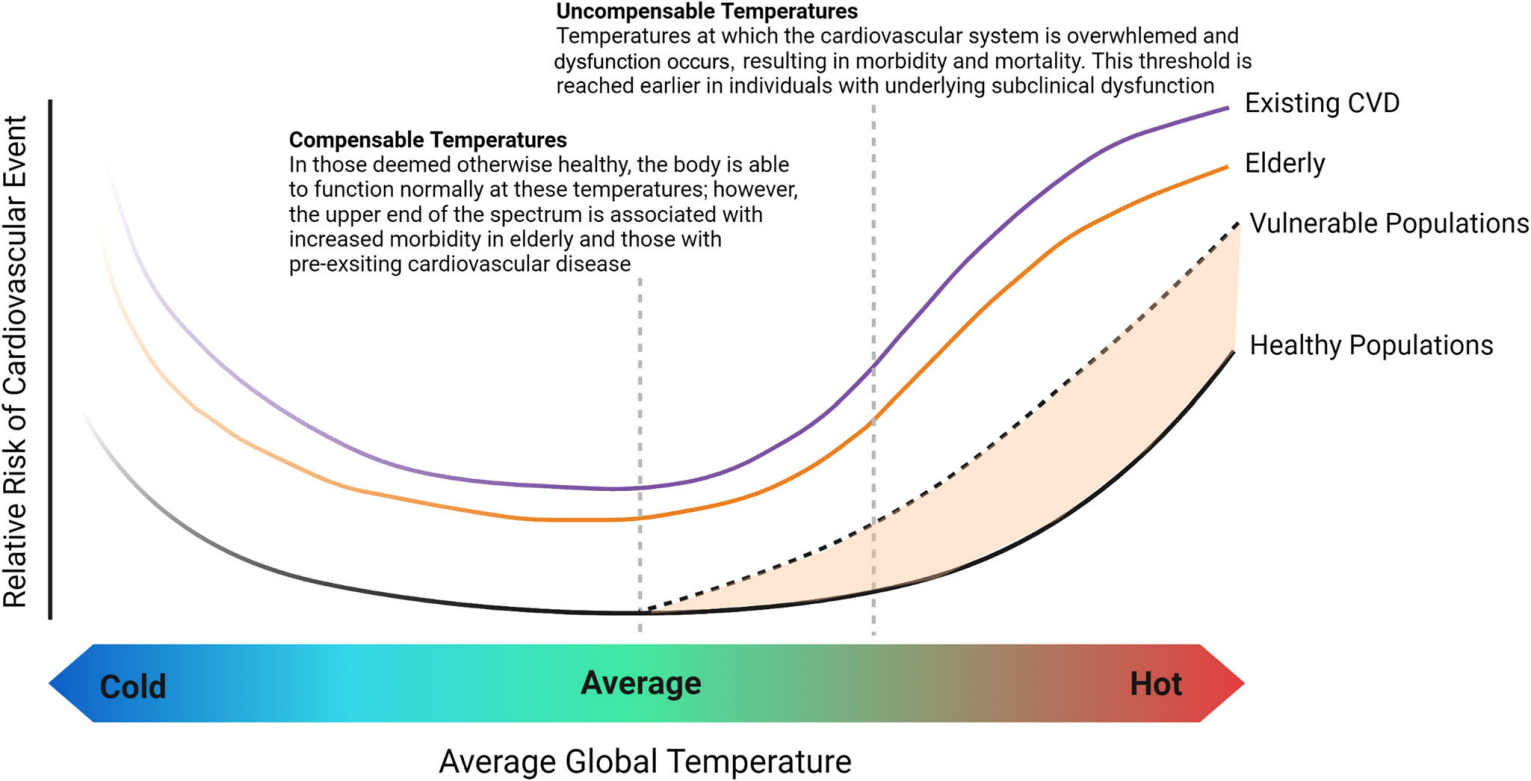
Relative risk of cardiovascular morbidity and mortality with exposure to increasing global temperatures. Thresholds for compensable and uncompensable thermal tolerance are altered by the presence of disease and dysfunction, for example, elderly populations (orange line) or cardiovascular disease populations (purple line). With rising global temperatures and greater severity of thermal extremes, the proportion of vulnerable populations increases (dotted line and shaded area). This cardiovascular risk may extend to apparently healthy populations, such as those born preterm. Created with BioRender.com.

High‐temperature days increase mortality by exacerbating pre‐existing health conditions (Campbell et al., 2018). The escalating frequency and severity of climate extremes is increasing this risk of morbidity and mortality in our vulnerable populations. At the same time, this climatic trend is interacting with societal health (e.g., ageing population, obesity) and infrastructure (e.g., intensification, pollution) to lower the threshold for vulnerability, such that the ‘at‐risk’ population is expanding to include less severe risk factors (Khraishah et al., 2022; K. R. Smith et al., 2014; Figure 1). Cardiovascular disease (CVD) and dysfunction remains the prominent driver (Casas et al., 2016), with more than 50% of adverse health events during high‐temperature days being of cardiovascular origin, whereas heat stroke and heat exhaustion contribute comparatively less (Campbell et al., 2018; Kang et al., 2016). Indeed, Danet et al. (1999) estimated as many as 10% of all myocardial infarctions may be due to fluctuations in the thermal environment. Given this tight association, the Intergovernmental Panel on Climate Change (IPCC) predicts that the main risk for morbidity across the first half of this century will be though climate‐induced exacerbation of health conditions in vulnerable populations (K. R. Smith et al., 2014).

CVD is a leading cause of morbidity and mortality worldwide, but symptoms frequently remain concealed until the cardiovascular system is stressed, as commonly occurs during thermal extremes (Casas et al., 2016). This perhaps explains the dramatic increase in CVD‐related hospital admissions during heat waves (Kim et al., 2017; Wang & Lin, 2014) and cold snaps (Hess et al., 2009; Shoraka et al., 2022), both acutely and in the days following exposure (Kang et al., 2016; Wang & Lin, 2014). Other factors, such as ageing (>60 years; Gravel et al., 2021; Holowatz & Kenney, 2010) and metabolic disease (Kenny et al., 2010; Wang & Lin, 2014) further enhance this cardiovascular risk in vulnerable populations. Among vulnerable population subgroups presenting with limited adaptive capacity, such as the elderly, heat‐ and cold‐stress may favour the occurrence of disease with early death (Liu et al., 2015; Shoraka et al., 2022). For instance, excess mortality during the 2003 European heat wave exceeded 10% in most countries across Europe (Robine et al., 2008), and heat illness during the North American heat wave rose 69 times above the same period in 2019 (Philip et al., 2021). While not examined by Robine et al. (2008) or Philip et al. (2021), it is likely that vulnerable populations with comorbidities contributed to this excess mortality. As cardiovascular dysfunction and disease is a leading comorbidity, cardiovascular events are likely the primary cause of excess mortality in these heat waves (Casas et al., 2016). However, when examining the effects of thermal extremes on excess morbidity and mortality, it is of interest not only to examine the response of vulnerable populations to temperature extremes, but also to understand the mechanisms that impair vulnerable subpopulations (Wang & Lin, 2014).

Those born preterm are one such vulnerable population. Worldwide, preterm birth accounts for 15 million live births annually (Lewandowski et al., 2020). Therapeutic advancements including antenatal corticosteroids, high frequency ventilation and surfactant therapies have allowed unprecedented numbers of preterm infants to survive (Crump, 2020), and at gestational ages previously thought unviable (<25 weeks’ gestation, full term 40 weeks’ gestation; Ancel et al., 2015). Despite improved survival rates, the life‐course burden of morbidity remains high in extremely preterm infants (Crump, 2020; Lewandowski et al., 2020). Across the spectrum of prematurity, preterm infants (<37 weeks’ gestation) are at an acute risk of neurological, cardiovascular and respiratory complications (Crump, 2020; McKinlay & Manley, 2019). However, cardiovascular and metabolic dysfunction continue beyond acute neonatal intensive care in a lifelong trajectory of cardiometabolic dysfunction (Crump, 2020; Lewandowski et al., 2020).

The risk of climate change‐induced exacerbation of health conditions in vulnerable populations highlights the need to understand the ways in which highly susceptible subpopulations are affected by extreme temperatures (Khraishah et al., 2022; Wang & Lin, 2014). As increasing numbers of preterm infants are surviving into adulthood it is of great importance that we understand how environmental temperature extremes may impact their lifelong health. The purpose of the current review is, therefore, two‐fold: first, to explore the mechanistic factors that make at‐risk populations vulnerable to thermal extremes, and second, given the extensive evidence demonstrating a lifelong elevated preterm‐associated CVD risk, to highlight those born preterm as a vulnerable population worth greater consideration in the climate change context.

## 2 COMORBIDITIES AND MODIFIERS OF THERMOREGULATION

**FIGURE 2:**
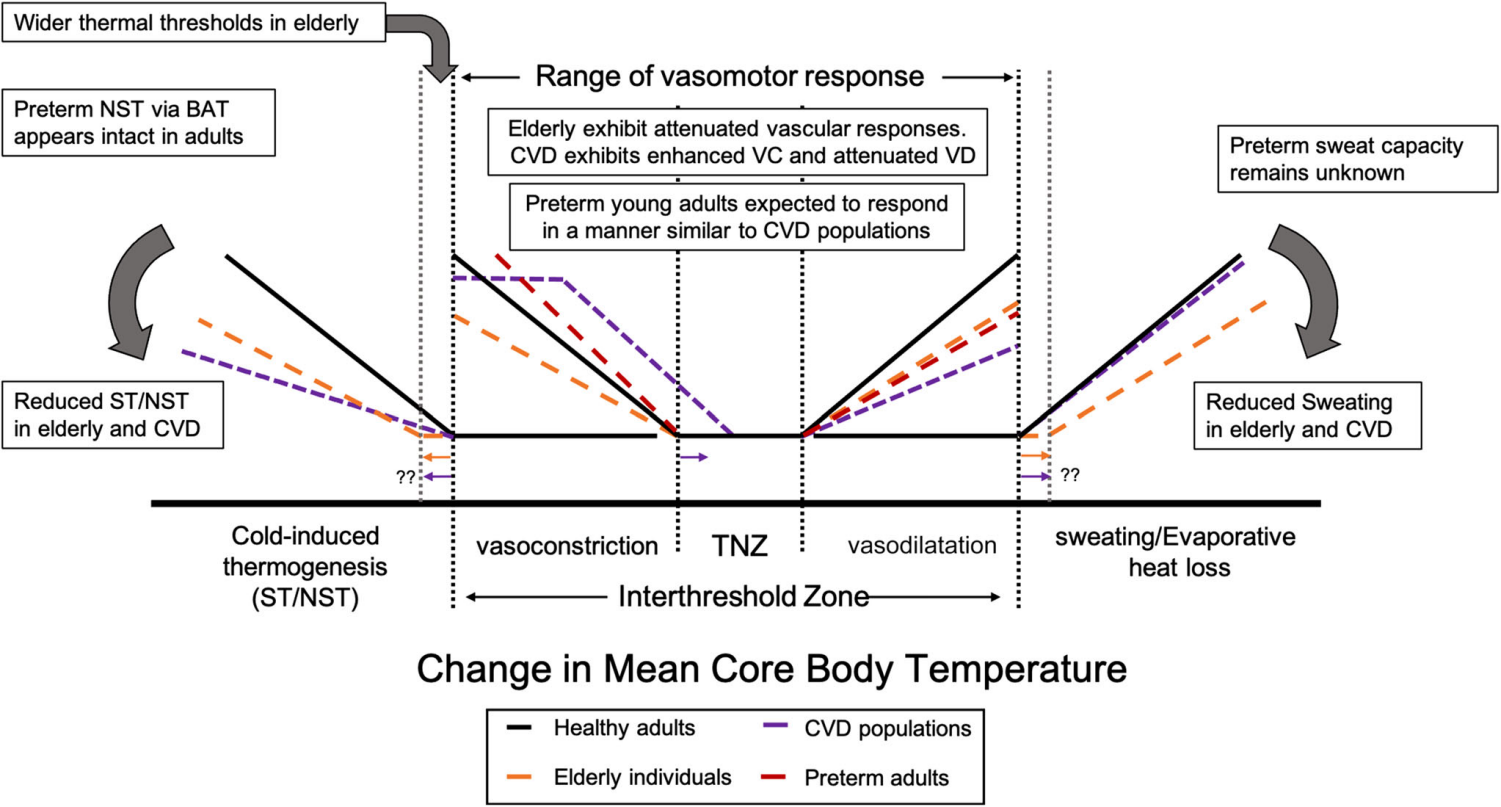
Thermoeffector responses in elderly, cardiovascular disease, and ex‐preterm populations during deviations in *T*_C_ by the presence of disease and dysfunction. Elderly: vasomotor response ↓. VC↓, due to: (1) ↓ sympathetic signalling (Greaney et al., 2015); (2) ↓ transmitter synthesis and axon release (almost sole reliance on NAd; Thompson & Kenney, 2004); and (3) ↓ post‐junctional responsiveness (Frank et al., 2000; Thompson & Kenney, 2004). In heat, absolute Sk.BF ↓ (H. L. Martin et al., 1995), responsiveness ↓ (Holowatz & Kenney, 2010) and hepato‐splanchnic BF redistribution ↓ (Gravel et al., 2021). CIT: cold perception ↓ (Florez‐Duquet & McDonald, 1998; Watts, 1971), ↓ Sk.M and therefore ↓ ST (Grimby & Saltin, 1983), ↓ BAT activity (Ruiz et al., 2018; Yoneshiro et al., 2011) and ↓ CIT (Florez‐Duquet & McDonald, 1998). Sweat response ↓, ↓ E_sw_ (Balmain, Jay et al., 2018), sweat gland atrophy, sensory nerve denervation and wider thermal thresholds (Guergova & Dufour, 2011; Inoue, 1996; Inoue et al., 1999). CVD populations: vasomotor response: dysregulation of CVS in CVD ↑'s VC (Foëx & Sear, 2004; Greaney et al., 2017; Holowatz & Kenney, 2007), and ↓'s VD (Cui et al., 2005; Kenny et al., 2010), due to ↑ SNA, ↓ endothelial VD mediators, ↓ capillary recruitment, rarefaction and fibrosis of vasculature (↓ NOS, ↔ ET‐1; Foëx & Sear, 2004; Holowatz & Kenney, 2007). Experimental evidence limited in CVD, but CIT ↓, ↓ Sk.M. and therefore ↓ ST (Tyrovolas et al., 2020), BAT activity inversely correlated with BMI and visceral adiposity, and UCPs ↓ in heart failure (Laskowski & Russell, 2008). CVD sweat response ↔ (Balmain, Sabapathy et al., 2018; Cui et al., 2005). BV ↓ and therefore ↓ E_sw_. Preterm adults: the preterm vasomotor responses are speculative as there are no published investigations in preterm thermovascular responses. Preterm adults exhibit microvascular rarefaction (Johansson et al., 2005; Lewandowski et al., 2015), endothelial dysfunction (Skilton et al., 2011), impaired NO sensitivity (Gray et al., unpublished results) and likely ↓ VD. ↑ VC driven by increased circulating catecholamines (Johansson et al., 2007) and narrowed arteries (Jiang et al., 2006; Schubert et al., 2011). CIT: BAT deposition appears comparable to term adults (Kistner et al., 2018). Abbreviations: BAT, brown adipose tissue; BF, blood flow; BV, blood volume; CIT, cold‐induced thermogenesis; E_sw_, evaporative heat loss from sweating; ET‐1, endothelin 1; NAd, noradrenaline; NOS, nitric oxide synthesis; Sk.M., skeletal muscle; NST, non‐shivering thermogenesis; Sk.BF, skin blood flow; SNA, sympathetic nerve activity; ST, shivering thermogenesis; *T*_C_, core temperature; TNZ, thermoneutral zone; UCP, uncoupling protein; VC, vasoconstriction; VD, vasodilatation.

While humans utilize a multi‐organ approach to maintain homeothermy, the cardiovascular system is the ‘linchpin’ by which homeothermy is maintained. With deviations from thermoneutral conditions, thermoeffectors are recruited in a co‐ordinated manner relative to their physiological cost. These consist of adjustments in vascular tone and thermal behaviours (e.g., seeking shade or shelter) followed by autonomic thermoeffectors (e.g., sweating or shivering/non‐shivering thermogenesis) (Schlader et al., 2018; C. J. Smith & Johnson, 2016; Figure 2). Whereas recruited thermoeffectors (i.e., sweating or shivering) may supersede the role of the cardiovascular system in terms of heat loss or heat conservation, they are accompanied by changes in vasomotor tone (Schlader et al., 2018). Schlader et al. (2018) demonstrated that even behavioural responses coincide with, but do not attenuate, changes in vasomotor tone. Cutaneous vasodilatation is important in thermoregulatory sweating, providing both the heat required for evaporation and the blood plasma necessary for sweat gland function (C. J. Smith & Johnson, 2016). Sweating further exacerbates cardiovascular strain under heat stress in its use of provided blood plasma. Indeed, it has been long established that a decrease in body weight of more than 2% as a result of sweating places severe demands on both the cardiovascular and thermoregulatory systems (Baker, 2019).

With ageing (>60–65 years), the physiological capacity to tolerate thermal stress declines (Balmain, Sabapathy et al., 2018) (Figure 2). The age‐related changes in thermoregulatory responses include attenuated vascular reactivity and elevated cardiac strain (Holowatz & Kenney, 2010), augmented pressor responses (Hess et al., 2009; Tochihara et al., 2021), and wider deviations in core body temperature (*T*_C_) (Guergova & Dufour, 2011; Holowatz & Kenney, 2010). This has been demonstrated both experimentally (Hess et al., 2009; Tochihara et al., 2021) and epidemiologically (Kim et al., 2017; Wang & Lin, 2014). While lifestyle and environmental factors can modify this decline in function, they cannot completely alleviate or ameliorate it (Tochihara et al., 2021). Collectively, the reduced tolerable capacity of senescent systems in elderly individuals is ultimately borne out in their excess deaths during thermal extremes.

The presence of CVD dramatically alters vascular reactivity, but more importantly it increases stress on the heart (Figure 2). Sympathetic overactivity enhances the constrictory state at rest, elevating afterload and blood pressure (BP) (Greaney et al., 2017; Ikaheimo, 2018). While the exact mechanisms are unknown, sustained low‐grade inflammation, endothelial dysfunction and autonomic dysregulation are implicated (Buford, 2016; Foëx & Sear, 2004). Endothelial dysfunction from chronically high BP impairs eNOS, without impairing production of endothelin‐1, a potent constrictor (Buford, 2016; Foëx & Sear, 2004). As such, the progression of CVD augments cooling‐invoked vasoconstriction, and impairs active vasodilatation. Stiffening of conductance vessels accentuates the pulsatility of blood flow, damaging endothelium, impairing dilatation, raising systolic and diastolic BP, and thereby afterload, aggravating cardiovascular workload and strain (Greaney et al., 2017; Ikaheimo, 2018). An additional consideration for this population is the role of medications in treating overt disease (e.g., beta blockers limiting heart rate) and blunting compensation (reviewed elsewhere). In healthy individuals, the increased myocardial workload elicits a sympathetically mediated dilatation in coronary vessels to support the myocardial demand for oxygen. However, diseased coronary vessels appear to paradoxically constrict, causing myocardial ischaemia and limiting cardiac workload (Nabels et al., 1988; Zeiher et al., 1989). This ischaemia can contribute to damage or scarring of the myocardium, and possibly myocardial infarction (Ikaheimo, 2018). Additionally, attenuated and ultimately reduced heat‐induced vasodilatation in CVD increases reliance on evaporative heat loss to limit the rise in *T*_C_; this can further exacerbate myocardial oxygen demand, limiting the capacity of cardiac output to accommodate for the change in haemodynamics.

CVD demonstrably reduces the cardiovascular capacity to tolerate stressors. This cardiovascular impairment is worsened by cumulative years of disease burden and is readily observed in the cardiovascular‐related hospital admissions during heat waves and cold snaps, particularly in elderly individuals who may possess latent or overt cardiovascular dysfunction (Kang et al., 2016; Liu et al., 2015). Moreover, societal factors, such as urbanisation (e.g., heat islands; Campbell et al., 2018) and pollution (Khraishah et al., 2022), enhance the severity of climatic events, further exacerbating the risk to vulnerable populations. As such, for those presenting with limited adaptive capacity, heat or cold stress may favour the onset of disease with early death (Liu et al., 2015). Indeed, those born preterm are one such sub‐population that possess a life‐long ‘latent’ risk – not the least of which is elevated BP – which may be uncovered by the stress of thermal extremes. In light of the indisputable rise in global temperatures, the preterm‐born population is one sub‐population that highlights our need to understand the health risk of climatic events.

## 3 PRETERM BIRTH AND CARDIOVASCULAR RISK ACROSS THE LIFESPAN

**FIGURE 3:**
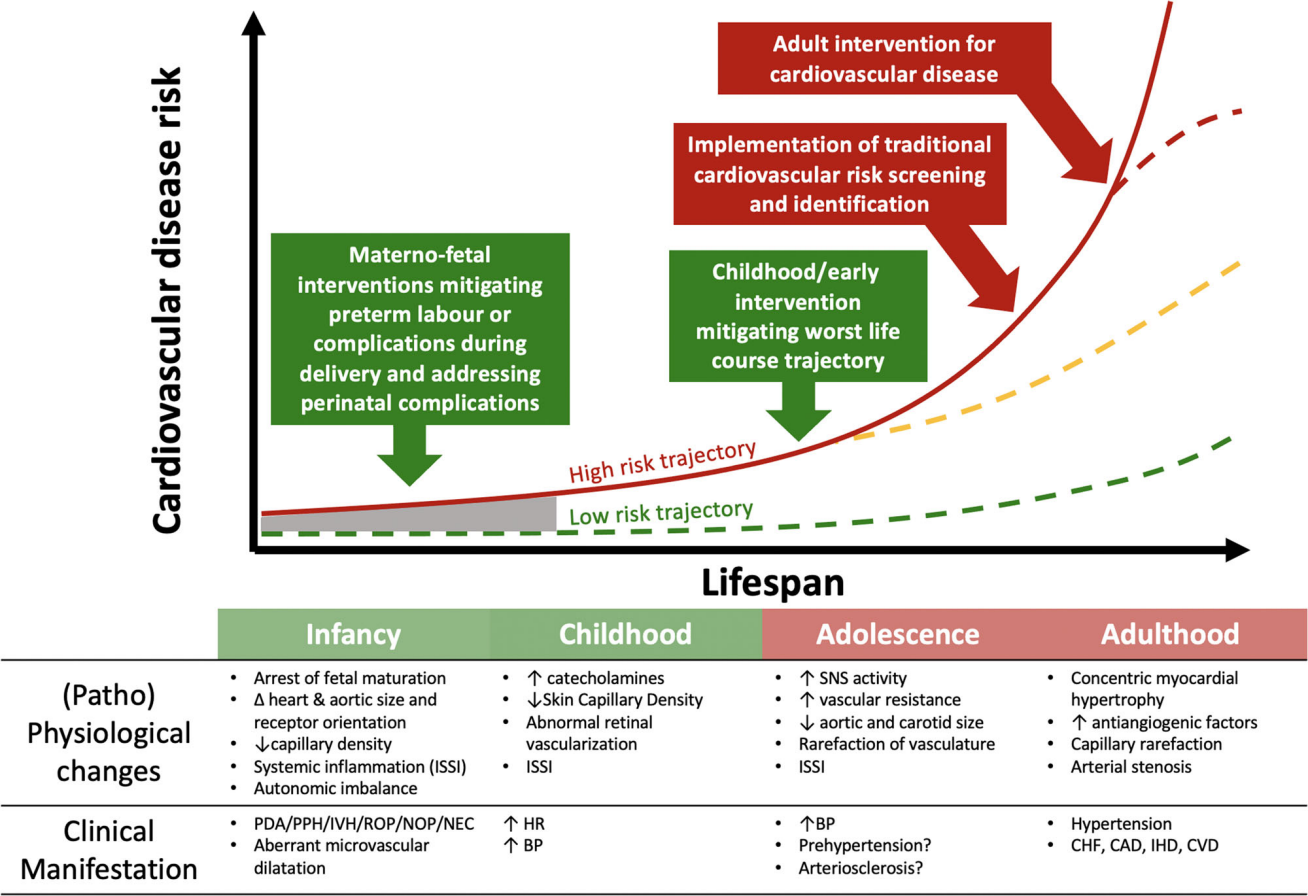
Life course view of preterm CVD risk. Risk of CVD increases in a non‐linear trajectory due to cumulative progression of dysfunction and disease from perinatal, lifestyle‐induced or other challenges. Prematurity (grey region) elevates the starting trajectory for CVD away from term‐born low‐risk trajectory. Early interventions may lower this high‐risk trajectory, whereas adult interventions of overt CVD have limited benefits. No treatment exists to lower preterm‐born individuals to a low‐risk trajectory. Figure adapted from Hanson and Gluckman (2014). Abbreviations: BP, blood pressure; CAD, coronary artery disease; CHF, congestive heart failure; CVD, cardiovascular disease; IHD, ischaemic heart disease; ISSI, intermittent or sustained systemic inflammation; NEC, necrotising enterocolitis; NOP, nephropathy of prematurity; PDA, patent ductus arteriosus; PPH, persistent pulmonary hypertension; ROP, retinopathy of prematurity; SNS, sympathetic nervous system.

Events that alter the normal trajectory of early life development have profound implications for health and well‐being extending throughout life (Crump, 2020). Of the 15 million live preterm births worldwide annually, ∼85% are moderate‐to‐late preterm deliveries (32–37 weeks), with very and extremely preterm deliveries (<32 weeks) accounting for ∼15% (Lewandowski et al., 2020). Sufficient evidence now exists implicating preterm birth as an independent risk factor for CVD; while this cardiometabolic risk is inversely proportional to gestational age, it remains even for late preterm infants (Crump, 2020). Preterm‐associated disease formation consistently occurs earlier than in the general population due in part to cessation of fetal maturation (Lewandowski et al., 2013; Schubert et al., 2011), systemic inflammation (Humberg et al., 2020) and accelerated ageing of systems (Prior & Modi, 2020). Some of these changes are observable from birth (Cohen et al., 2007, 2008; Stark et al., 2008), whereas others become apparent across the lifespan. Dysfunction observed in childhood include pulmonary vascular disease (Naumburg & Soderstrom, 2019), arterial narrowing (Jiang et al., 2006; Schubert et al., 2011), and abnormal vascularisation (Bonamy et al., 2007; Hellström et al., 1998). In adulthood, higher rates of cardiometabolic dysfunction and disease have been widely observed (e.g., hypertension, diabetes mellitus, heart failure and ischaemic heart disease (Crump, 2020; Lewandowski et al., 2020). Despite efforts to reduce the harm of acute therapies on long‐term health outcomes of preterm‐born individuals (e.g., treatment of circulatory compromise, postnatal corticosteroids; McKinlay & Manley, 2019), treatments to address their long‐term cardiovascular risk remain scarce (Lewandowski et al., 2020). As such, those born preterm are a burgeoning population who carry an increased risk of CVD throughout life (Crump, 2020; Figure 3).

While it is clear that at‐risk populations experience the most detrimental effects during heat waves and cold snaps, the specific vulnerabilities of subpopulations require further attention in order to minimise excess morbidity and mortality (Wang & Lin, 2014). The preterm risk of CVD is well established, though their functional cardiovascular capacity remains less well‐known (Table 1). The risk of both age‐ and CVD‐related morbidity and mortality from thermal extremes is also well established, as discussed above (K. R. Smith et al., 2014). Given that even apparently healthy elderly individuals carry this increased burden of risk, this then implicates preterm‐born individuals, who despite appearing healthy in their early adulthood, carry underlying systemic cardiovascular dysfunction (Huckstep et al., 2018), and exhibit accelerated ageing (Prior & Modi, 2020). However, those born preterm have not been examined in relation to increased cardiovascular risk and thermal extremes. Here, we will attempt to connect the dots.

**TABLE 1:** The cardiovascular response to physiological stressors in preterm‐born individuals across the lifespan.

Reference	Subjects	Stressors	Measured variables	Results	Conclusion
**Infancy**					
Cohen et al. (2007)	12 healthy term (38–42 wks GA) and 12 preterm infants (27–34 wks GA) and 10 preterm infants diagnosed with BPD (23‐33 wks GA); examined 2–5 days post birth (terms) and at 36 and 40 wks postmenstrual age	Hypercapnia: 4 min, 4% CO_2_ @10 l min^−1^	ECG, BP, $T_{c,O_{2}}$, $T_{c,CO_{2}}$, $S_{{\mathrm{aO}}_{2}}$, respiration	BP response to CO_2_ was exaggerated at 36 wks but normalised at 40 wks. HR response was phase delayed compared to terms. BP response was depressed in severe BPD	Preterm infants had altered HR and occasionally altered pressor responses
Cohen et al. (2008)	29 term control (40 ± 1 wks GA), 18 term exposed to maternal smoking (39 ± 2 wks GA), 15 preterm‐AGA (31 ± 2 wks GA), 16 preterm‐SGA (32 ± 2 wks GA)	Hypercapnia: 4 min, 4% CO_2_. Orthostatic tolerance test	ECG, BP, $T_{c,O_{2}}$, respiration (CO_2_ test alone)	Ventilatory response to 4% CO_2_ was depressed in smoke‐exposed and preterm infants. Smoke‐exposed infants had exaggerated BP and HR response and preterm infants had minimal HR response. The tilt test produced excessive BP response in smoke‐exposed and preterm infants but normal HR response	Preterm infants and those exposed to maternal smoking alters cardiovascular control from birth
Moss et al. (1963)	74 full term and 26 preterm infants (unspecified GA), examined between 1 and 77 h PNA (B/W range: 1.2–4.5 kg). Cold pressor performed on 29 full term and 17 preterm infants. Tilt test performed on 27 full term and 23 preterm infants	Cold pressor test: exposure of 1 foot to 4–5°C water for 1 min. Orthostatic tolerance test	Descending aortic pressure (via umbilical artery cannulation), derived HR	HR, SBP and DBP increased similarly in both term and preterm infants although preterm HR and BP were lower. HR, SBP and DBP responses were similar between term and preterm infants, though preterm infants were consistently lower in all measures	Both term and preterm infants demonstrated a capacity to respond to cardiovascular challenges
Rodrigues & Guinsburg (2013)	36 preterm infants (28–32 wks GA) without congenital or postnatal complications, assessed during diaper change at 72 h, 7 days, 14 days, 21 days and 28 days PNA	Pain during painless procedures (diaper change)	HR, $S_{{\mathrm{aO}}_{2}}$, behavioural measures of pain (BIIP, NIPS, PIPP)	Analysis of pain profile did not indicate increased pain presence or pain scores during procedures	Preterm infants are not sensitised to non‐painful stimuli following standardised NICU care
**Childhood**					
Hermann et al. (2006)	19 preterm‐born children (28.6 ± 2.0 wks GA; 11.4 ± 1.3 yrs; *n* = 9 female), 20 term children (12.0 ± 1.2 yrs; *n* = 6 female) with NICU care, and 20 term without (11.2 ± 1.8 yrs; *n* = 10 female), exposed to heat pain stimulation	Sensitization to heating at pain threshold at thenar and trigeminal skin sites	*T*_sk_ at perceived pain threshold following primary heat exposure	Preterm and term children exposed to NICU experienced significant sensitisation to heat at the thenar site, but significantly less habituation to heat at the trigeminal site compared to controls	Exposure of preterm and term infants to painful procedures in NICU alters pain pathways, increasing sensitisation to heat pain in childhood
Johansson et al. (2007)	39 preterm (26.6 ± 2.0 wks GA), 29 SGA term (39.3 ± 1.4 wks GA), and 37 control term (39.6 ± 1.0 wks GA) children (9.6 yrs) underwent an orthostatic tolerance test and mental stress test	Orthostatic tolerance test. Mathematical mental stress test, subtracting a consistent number to 0	HR, SBP, DBP and urinary catecholamines	Catecholamines were significantly raised in preterm and SGA children as well as HR at rest, but not SBP or DBP. HR was significantly elevated by mental stress in preterm and SGA children	Sympathoadrenal activity is raised in childhood, and cardiovascular control is differentially regulated in preterm and SGA children
Lowe et al. (2016)	12,781 children 7 y/o were provided activity monitors for 7 days. 79 children were born at GA 24–32 wks, 119 were 33–34 wks GA, 275 at 35–36 wks GA, and 5949 were term‐born controls	PA	Accelerometery	Boys born <32 wk GA, but not other GA groups, had significantly reduced physical activity compared to term controls (equating to 9 min less/day)	Boys born <32 wks GA, participate less in PA equating to 1 h/wk less than term children
L. J. Smith et al. (2008)	126 children born at an average of 27 wks GA, and 34 term‐controls underwent testing at 10 y/o. Testing included lung function and fitness	6‐min walk and 20 m shuttle run test	TLC, VLC, RV, FRC, ${\dot{V}}_{O_{2}\mathrm{peak}}$, and HR	All respiratory measures were reduced, including TLC, VLC and FRC. Exercise capacity was approximately half that of term children	Children born preterm had reduced exercise capacity, that was not fully explained by their reduced respiratory capacity
Walker et al. (2009)	43 extremely preterm children (<25+6 wks GA) and 44 term control underwent sensory and cognitive testing	Thermosensation from cold to hot with baseline at 32°C adjusted at 1°C min^−1^ (range: 10–50°C)	*T*_sk_ of thenar skin, and perception of temperature thresholds	Sensation of temperature across all modalities was significantly reduced in preterm children. Neonatal surgery also decreased sensitivity but cognition was similar among preterms	Preterm born children have decreased general thermal sensitivity that may be due to central modulation of nociceptive pathways
Welsh et al. (2010)	38 extremely preterm children (27 females; < 25 wks GA), and 38 term controls underwent pulmonary function and exercise testing	Peak exercise test	Accelerometery, HR, SBP, DBP, $S_{{\mathrm{pO}}_{2}}$, *f*_B_, RER, *V*_T_, ${\dot{V}}_{O_{2}}$, work/kg	*f*_B_ was significantly faster in preterms at rest, but lower during peak exercise, along with ${\dot{V}}_{O_{2}}$, *V*_T_ and work/kg at peak exercise	Preterm children had lower ${\dot{V}}_{O_{2}\mathrm{peak}}$, and different respiratory adaptations not explained by PA
**Adolescence**					
Haraldsdottir et al. (2018)	20 term‐born (11 female) and 12 preterm‐born (13 female) adolescents (<36 wks GA), at 12–14 y/o	Graded exercise testing on a cycle ergometer: 25 W increments in 2 min intervals	${\dot{V}}_{O_{2}}$, HR, HRV, PA	${\dot{V}}_{O_{2}\max}$ was significantly lower in preterm‐born adolescents. LF HRV was significantly higher. HR recovery was significantly longer	Preterm‐born adolescents had autonomic dysfunction and worse HR recovery following exercise
Clemm et al. (2014)	34 preterm‐born (<28 wks GA; 16 female) and 33 term‐born adolescents (16 female) examined at 17.5 ± 1.2 y/o and 17.8 ± 1.2 y/o then 24.7 ± 1.2 y/o and 25.1 ± 1.2 y/o. Neonatal BPD: *n* = 17 mild, *n* = 8 moderate/severe	Exercise ramp test: Bruce protocol	${\dot{V}}_{O_{2}\mathrm{peak}}$, FEV_1_, FVC, HR, self‐reported PA	Reported activity was similar between groups. ${\dot{V}}_{O_{2}\mathrm{peak}}$ was not significantly different (40.7 and 44.2 ml kg^−1^ min^−1^) and not correlated to neonatal factors but rather PA levels	Exercise capacity of preterm‐born adolescents was within normal ranges and correlated with self‐reported PA
van Ganzewinkel et al. (2017)	412 preterm‐born (31.1 ± 2.5 wks GA) young adults (19 y/o) enrolled in this follow up study	Cold pressor test: forearms were immersed in 4–6°C water for 3 min	Pain Coping Questionnaire, pain threshold and tolerance	Female gender and NEC significantly reduced pain tolerance	The occurrence of NEC was the only determinant neonatal of lower pain threshold and pain tolerance in adulthood
**Adulthood**					
Haraldsdottir et al. (2019)	12 preterm‐born (28.5 ± 2.7 wks GA) and 16 term‐born (39.5 ± 0.6 wks GA) completed exercise testing at 26.9 ± 1.1 y/o and 25.6 ± 0.7 y/o	Graded exercise testing on a cycle ergometer under normoxic and hypoxic (12% O_2_) conditions	Self‐reported PA (G‐PAQ), HR, ${\dot{V}}_{O_{2}\max}$, *P*_max_, *T*_max_, RER	PA was similar between groups. Preterms had significantly lower. ${\dot{V}}_{O_{2}\max}$ (34.9 ± 9.3 ml kg^−1^ min^−1^ vs. term: 45.8 ± 8.7 ml kg^−1^ min^−1^, normoxia), *P*_max_, *T*_max_ and HR recovery following challenge	HR recovery is significantly slower in those born preterm immediately following maximal exercise indicating autonomic dysregulation
Huckstep et al. (2018)	47 preterm‐born (32 ± 3.2 wks GA) and 54 term‐born adults participated in cardiopulmonary exercise testing in two cardiovascular challenges	CPET testing on a cycle ergometer: 15 W increments to exhaustion from 35 W or 75 W based on fitness. Submaximal exercise in 3 min intervals of 40%, 60% and 80% of peak CPET testing	HR, BP, ${\dot{V}}_{O_{2}}$, RER, RPE, venous blood, echocardiography. Cardiac output derived from stress echocardiography	Preterm‐born adults had greater LV wall thickness and smaller chamber size and higher HR at rest. RER achieved at peak exercise intensity was similar between groups. Ejection fraction, similar at rest reduced significantly compared to term‐born adults at 60% and 80% of CPET intensity. Cardiac output reserve was significantly reduced at 40%, 60% and 80% CPET intensity	Preterm‐born adults have altered LV structure and function at rest, which is exacerbated by exercise resulting in significantly impaired systolic response, decreased EF, and cardiac reserve appearing from mild exertion
Macdonald et al. (2021)	11 preterm born adults (27 ± 1 years; 29+3 wks GA) and 17 preterm‐born adolescents (13 ± 1 years; 28 ± 2 wks GA) compared to 11 young adults (26 ± 1 years; 40 ± 1 wks GA) and 18 adolescent (13 ± 1 years; 40 ± 1 wks GA) age‐match controls	Step test at 60 steps/min, 70% of predetermined ${\dot{V}}_{O_{2}\max}$	HR, SV, right ventricle volumetry, ${\dot{V}}_{O_{2}\max}$	SV response to exercise was depressed, increasing reliance on HR. Cardiac output response to exercise was significantly reduced	Preterm subjects demonstrated impaired SV and right heart function with exercise, a response associated with many right heart diseases
Barnard et al. (2020)	10 preterm‐born (28 ± 1 wks GA) and 12 term‐born (>36 wks GA) adults participated in two normoxic and hypoxic (12% O_2_) prolonged graded exercise tests to maximum	Prolonged graded cycle test: 50 W start, with 10 W increments every 2 min, until failure to maintain >55 rpm	HR, BP, brachial artery pulse wave velocity	Preterm‐born adults had elevated pulse wave velocity alongside increased HR, SBP, PP throughout normoxic exercise. Hypoxia reduce DBP in preterm but not term adults	Preterm‐born adults exhibit significantly elevated BP during exercise

The following studies during infancy, childhood, adolescence and adulthood are by no means an exhaustive list, but are representative of literature at these life stages. Abbreviations: AGA, average for gestational age; BIIP, behavioural indicators of infant pain; BPD, bronchopulmonary dysplasia; B/W, birth weight; CPET, cardiopulmonary exercise testing; DBP, diastolic blood pressure; EF, ejection fraction; *f*_B_, breathing frequency; FEV_1_, forced expiratory volume in 1 s; FRC, functional residual capacity; FVC, forced vital capacity; GA, gestational age; HF, high frequency domain of HRV analysis; HRV, heart rate variability; LF, low frequency domain of HRV analysis; LV, left ventricle; NICU, neonatal intensive care unit; NIPS, neonatal infant pain scale; PNA, postnatal age; PIPP, premature infant pain profile; *P*_max_, maximum power; PP, pulse pressure; RPE, rating of perceived exertion; RV, residual volume; $S_{{\mathrm{pO}}_{2}}$/$S_{{\mathrm{aO}}_{2}}$, oxygen saturation; SBP, systolic blood pressure; SGA, small for gestational age; $T_{c,O_{2}}$/$T_{c,CO_{2}}$, transcutaneous concentration of carbon dioxide or oxygen; TLC, total lung capacity; *T*_max_, time to exhaustion; *T*_sk_, skin temperature; VLC, vital lung capacity;${\dot{V}}_{O_{2}\max}$/${\dot{V}}_{O_{2}\mathrm{peak}}$ maximal or peak oxygen consumption; *V*_T_, tidal volume; y/o, years old.

### 3.1 Cardiovascular (dys)function in preterm‐born individuals

Preterm birth per se is the single most pervasive and lasting materno‐fetal insult to preterm infants (Bavineni et al., 2019; Crump, 2020). Transition to extrauterine life is a period of an exceptionally dynamic and tightly coordinated physiological change, catalysed by changing materno‐fetal hormonal profiles, parturition with separation from the placenta, and the first breaths. However, premature transition profoundly disrupts normal fetal maturation of cardiovascular, metabolic and neural systems (Lewandowski et al., 2020; Prior & Modi, 2020). In the cardiovascular system, this can be observed in abrupt maturation of cardiomyocytes, characterised by cessation of myocyte proliferation, reduced ventricular size and myocyte number, increased collagen deposition and smaller relative internal ventricular diameters – key cardiac structural elements which at the time of term birth are largely set for life (Lewandowski et al., 2013). Growth disruption also extends across the vasculature resulting in narrowed arteries (e.g., aortic, coronary, popliteal and brachial arteries), an anti‐angiogenic state and disorganised microvasculature (Lewandowski et al., 2015; Schubert et al., 2011). Poor circulatory adaptation drives much of the clinical dysfunction observed in the preterm neonate (Knobel et al., 2009; Stark et al., 2008), but it remains a key driver of lifelong cardiovascular decompensation.

While the level of postnatal cardiovascular catch‐up development, growth and functional recovery is unknown, the persistence of cardiovascular dysfunction in later life argues that it is limited. In childhood, this trajectory of cardiovascular dysfunction manifests in the form of elevated BP, heart rate and circulating catecholamines (Bonamy et al., 2007; Johansson et al., 2007), as well as reduced capillary density, and abnormal retinal and cutaneous vascularisation (Bonamy et al., 2007; Hellström et al., 1998). Indeed, many studies have reported the persistence, and further deterioration of, arterial dysfunction throughout infancy (Schubert et al., 2011; Tauzin et al., 2014), childhood (H. Martin et al., 2000), adolescence (Bonamy et al., 2005; Johansson et al., 2005) and adulthood (Hovi et al., 2011; Tauzin et al., 2014).

While the association between preterm‐born individuals’ arterial function and atherosclerosis formation remains uncertain, the effects on BP and afterload remain clear precursors to CVD. Indeed, elevated BP has both upstream (eccentric myocardial hypertrophy) and downstream effects. Both macro‐ and microvascular dysfunction contribute to elevated BP, with capillary rarefaction caused by, and further contributing to, sustained elevations in BP through their effect on systemic vascular resistance (Barnard et al., 2020; Lewandowski et al., 2015). Persistently elevated BP damages the endothelial lining, contributing to atherosclerotic plaque formation in major vessels and impairing eNOS throughout the vasculature (e.g., via oxidative stress‐mediated endothelial damage, and capillary rarefaction; Bavineni et al., 2019). Capillary rarefaction, and the associated antiangiogenic state demonstrated in preterm‐born adolescents and adults, has been hypothesised to be a lasting effect from birth and causative in the preterm‐risk of hypertension (Kistner et al., 2002; Lewandowski et al., 2015).

Systemic inflammation and sympathetic hyperactivity may also play a role in the persistence of cardiovascular dysfunction from infancy (Bavineni et al., 2019; Humberg et al., 2020). Preterm birth disrupts development of autonomic maturation, leaving the parasympathetic arm to mature postnatally (De Rogalski Landrot et al., 2007; Patural et al., 2008). A functional interdependence has been hypothesised to form between the hyperadrenergic state and systemic inflammation, as preterm populations in the neonatal period ‘collect’ risk factors for chronic inflammation (e.g., respiratory inflammation, sepsis, enterocolitis), which alongside the NICU environment (e.g., bright lights, painful procedures, excess noise) may additionally interfere with maturation of autonomic control (Patural et al., 2008; Yiallourou et al., 2013). This relationship between systemic inflammation and sympathetic hyperactivity is causative in the aetiology of more traditional CVD (Buford, 2016; Humberg et al., 2020). In preterm infants, such factors may cement the autonomic imbalance beyond the neonatal period, as the immaturity of the descending modulating pathways appear to potentiate the neonatal stress response, both acutely and chronically, via neuroplastic mediators (Rodrigues & Guinsburg, 2013; van Ganzewinkel et al., 2017; Walker et al., 2009). This could further explain why maturation of parasympathetic nervous system (PNS) activity lags so far behind sympathetic maturation (PNS maturation suppressed in magnitude 2‐ to 3‐fold compared to term‐born counterparts at 6 months; Yiallourou et al., 2013), and may further compromise heart rate recovery from stressors throughout life (tilt test in infancy, Yiallourou et al., 2013; exercise in adults, Haraldsdottir et al., 2019).

Importantly, any postnatal ‘catch up’ occurs on a background of structural deterioration of the vasculature and endothelium alongside excess circulating catecholamines (Bonamy et al., 2005; Johansson et al., 2005; Kistner et al., 2002), leading to increased shear stress, oxidative stress and inflammatory markers (Bavineni et al., 2019). Systemic inflammation is further aggravated by the presence of other chronic preterm‐specific dysfunction, including obesogenic factors and insulin resistance, which exacerbate and accelerate CVD formation (Humberg et al., 2020). This system‐wide cardiovascular dysfunction associated with all abbreviated gestations (<37 weeks) necessarily affects the capacity to respond to stressors, such as psychosocial stress, exercise and thermal extremes *even in the absence of overt disease*. This is due to the cessation of fetal maturation, inflammation and accelerated ageing incurred as a result of preterm birth and early postnatal life. Early onset of CVD in those born preterm increases the cumulative years of disease burden, enhancing their risk in the face of thermal extremes.

### 3.2 Thermal resilience in preterm‐born individuals

The response of preterm‐born individuals to physiological stress has been examined in infancy, childhood, adolescence and adulthood (Table 1). Though much of this focus has been on respiratory capacity and exercise tolerance following extreme preterm birth (L. J. Smith et al., 2008), as well as orthostatic or CO_2_ tolerance (Cohen et al., 2007, 2008) and potentiation of pain responses (Rodrigues & Guinsburg, 2013; van Ganzewinkel et al., 2017), inferences can be made regarding the response to environmental extremes. During the preterm perinatal period, cardiovascular and thermoregulatory instability (particularly of aberrant dilatation and deficient brown adipose tissue) have been well documented. Indeed, preterm infants are considered transiently poikilothermic, with this resolving in infancy (Knobel et al., 2009). The capacity of preterm‐born individuals to adequately thermoregulate beyond the neonatal period has not been examined, and as such the level of postnatal catch up is unknown. However, troubling allusions include poor thermosensitivity across the entire thermal spectrum and altered pain sensitivity in childhood (Hermann et al., 2006; Walker et al., 2009), as well as system‐wide cardiovascular dysfunction which is tightly interrelated with thermoregulatory capacity.

Cardiovascular responses to exercise stress appear to be reduced in healthy preterm‐born children, adolescents and adults in comparison to their term counterparts (L. J. Smith et al., 2008; Welsh et al., 2010; Table 1). Exercise capacity is already significantly reduced in childhood (∼50% of term counterparts; L. J. Smith et al., 2008); 20% reduced ${\dot{V}}_{O_{2}\mathrm{peak}}$, (Clemm et al., 2014), and is further impaired by neonatal bronchopulmonary dysplasia (Welsh et al., 2010). In adulthood, this disparity in exercise capacity remains (10% reduced ${\dot{V}}_{O_{2}\mathrm{peak}}$ in 18–25‐year‐olds; Clemm et al., 2014), with prematurity correlated with sedentary behaviour (Lowe et al., 2016). Huckstep et al. (2018), using an exercise stress test in young adults observed that while cardiac compensation in preterm‐born adults occurred at rest, upon performing exercise at 60% capacity, ejection fraction reduced 6.7% from their term controls, and further declined to 7.3% by 80% of exercise capacity. This was matched by a significant ∼50% reduction in cardiac reserve from 40% of exercise capacity (Huckstep et al., 2018). Such findings are supported by evidence of increased aortic stiffness, and associated BP elevations, as well as attenuated stroke volume and cardiac output during exercise in preterm born adults (Barnard et al., 2020; Macdonald et al., 2021). In addition to poor performance, recovery from exercise stress may also be affected, with Haraldsdottir et al. (2019) reporting significantly lower ${\dot{V}}_{O_{2}\max}$ and slower heart rate recovery from maximal effort.

Parallels can be drawn between exercise capacity and thermal risk (Wilson et al., 2014). Preterm‐born individuals at all ages demonstrate reduced exercise capacity, altered cardiac response and impaired recovery. Cardiac stress remains the dominant cause of morbidity and mortality during thermal extremes in elderly and CVD populations (Campbell et al., 2018; Kang et al., 2016), and is likely of greatest risk in preterm populations. Elderly hearts exhibit attenuated changes in cardiac output and elevated cardiac strain during heat stress (Gravel et al., 2021). Similarly, impaired cardiac output and ventricular function are present in preterm‐born adults under exercise stress (Huckstep et al., 2018; Macdonald et al., 2021). Like those with CVD, preterm populations exhibit persistent systemic inflammation and autonomic dysregulation. This has been shown in CVD populations to augment vasoconstriction and impair vasodilatory capacity (Ikaheimo, 2018; Kenny et al., 2010). Capillary rarefaction and fibrosis have been demonstrated in CVD to impair heat responses (Buford, 2016; Foëx & Sear, 2004); the prevalence of rarefaction, system‐wide fibrosis and antiangiogenic factors in prematurity likely also impairs capillary recruitment and heat offload. Furthermore, sustained BP elevations and systemic inflammation damage the endothelial lining impairing nitric oxide release, and this may compound structural insufficiencies in prematurity. Finally, impaired thermosensitivity apparent in both elderly and preterm populations delays thermoregulatory action (Hermann et al., 2006; Walker et al., 2009), increasing the load of subsequent responses. It should be clear, then, that those born preterm share many characteristics that make elderly, and particularly those with CVD, vulnerable. More research is required to elucidate this educated speculation.

As perinatal medicine continues to evolve, enabling increasing numbers of even extremely preterm infants to survive, the focus must necessarily shift to accommodate consideration of long‐term cardiometabolic wellbeing alongside innovation needed to improve neonatal survival. However, technological innovations are likely to mitigate only the worst life‐course trajectory in those born preterm. Given this, the unknown long‐term risk of morbidity and mortality from thermal extremes is of major concern and further studies are clearly needed.

## 4 DISCUSSION AND PERSPECTIVES

The IPCC predicts that the major risk for morbidity and mortality from climate change across the first half of this century will be through exacerbation of health conditions in vulnerable populations (K. R. Smith et al., 2014). Given rising global temperatures continually setting new temperature records, and a four‐ to five‐fold increase in heat waves, the threshold for vulnerability is reducing (World Meteorological Organization, 2019). Excess hospital admissions and death due to climatic extremes are now routinely being documented (Kim et al., 2017; Robine et al., 2008; Shoraka et al., 2022; K. R. Smith et al., 2014). This necessitates wider investigation of potentially vulnerable populations, such as those born preterm, in the climate change context.

In this review, we have highlighted some of the common cardiovascular factors between elderly and CVD populations, and those born preterm. These include impaired thermosensitivity and impaired vasomotor reactivity with greater cardiac strain under physiological stress (Figure 2). While we are unable to draw direct comparisons in thermoregulatory capacity between these populations, the similarities in cardiovascular dysfunction are apparent. Those born preterm exhibit dysfunctional cardiovascular traits from an early age (Figure 3), and exhibit more cumulative years of disease burden than ‘traditional’ CVD populations who frequently manifest signs of CVD in mid‐to‐late adulthood. Overt cardiovascular dysfunction can be observed in preterm‐born adolescents and young adults during exercise stress (Table 1). This includes progressive cardiac impairment, increased pulsatility of blood flow and elevated BP during graded exercise (Barnard et al., 2020; Huckstep et al., 2018; Macdonald et al., 2021). Similar limitations are observed in the progressive deterioration associated with ‘traditional’ CVD.

Those born preterm are, therefore, a population worthy of investigation in the climate change context. While the majority of those born preterm survive into adulthood without major comorbidities, the structural changes incurred at birth remain. This is true not just for those born at the limit of viability but *all individuals born less than 37 weeks’ gestation*. Poor recognition of this risk in adulthood limits timely detection of dysfunctional traits that make this population at risk of CVD and of morbidity or mortality during thermal extremes. Researchers, policymakers and public health officials would benefit from further examination of physiological responses to thermal stress in these vulnerable populations (Khraishah et al., 2022).

## 5 RECOMMENDATIONS FOR FUTURE RESEARCH

There remains much to learn regarding the response to heat and cold stress in vulnerable populations. This includes, but is certainly not limited to, the well‐known CVD population. Those born preterm are uniquely at risk because of their early exposure to extrauterine life, which impacts nearly all bodily systems. Events that alter the trajectory of early life development and how this predisposes infants to increased risk of non‐communicable disease in later life remain poorly recognised outside the field of developmental origins. However, preterm birth represents a relatively common and profound disruption to developmental physiological processes which can have far‐reaching impacts. Due to this paucity of data, there is clearly a need for more research investigating the true risk in preterm‐born populations. As this is a heterogeneous group, with vast differences between gestational ages, all future studies must be couched in these terms. A list of future research avenues includes:

- Are preterm populations present in the excess morbidity and mortality data of thermal extremes? Countries with adequate neonatal records should be capable of examining this question.
- Is impaired thermosensation present in adulthood? Mechanistically, why is thermosensation impaired?
- What is the preterm response to heat and cold stress? Is this comparable to findings during exercise? And are these findings present in moderate‐ to late‐preterm born individuals?
- Does thermal stress expose altered cardiac function as exercise appears to?
- Could augmented systemic inflammation contribute to elevated risk in heat or cold?
- What is the preterm vasomotor response to heat or cold exposure? What of thresholds for thermoeffector function? How effective is their sweat capacity? What of shivering and non‐shivering thermogenesis?
- Does vulnerability to stress coincide with cardiovascular dysfunction in adulthood, or are adolescents or even children at risk during thermal stress? Does any vulnerability correspond to that of known populations, or does their early life present a unique vulnerability?

## AUTHOR CONTRIBUTIONS

Ryan Phillip Sixtus, Rebecca Maree Dyson, Mary Judith Berry: conceptualisation, funding acquisition. Ryan Phillip Sixtus: original draft. Ryan Phillip Sixtus, Rebecca Maree Dyson, Clint Gray, Mary Judith Berry: review and editing of manuscript. All authors have read and approved the final version of this manuscript and agree to be accountable for all aspects of the work in ensuring that questions related to the accuracy or integrity of any part of the work are appropriately investigated and resolved. All persons designated as authors qualify for authorship, and all those who qualify for authorship are listed.

## CONFLICT OF INTEREST

The authors declare that they have no conflicts of interest.
